# Large Deviations for Subcritical Bootstrap Percolation on the Erdős–Rényi Graph

**DOI:** 10.1007/s10955-021-02819-w

**Published:** 2021-10-14

**Authors:** Omer Angel, Brett Kolesnik

**Affiliations:** 1 Department of Mathematics, University of British Columbia, Vancouver, BC Canada; 2 Department of Mathematics, University of California, San Diego, San Diego, CA USA

**Keywords:** Bootstrap percolation, Phase transition, Random graphs, Large deviations, Discrete calculus of variations

## Abstract

We study atypical behavior in bootstrap percolation on the Erdős–Rényi random graph. Initially a set *S* is infected. Other vertices are infected once at least *r* of their neighbors become infected. Janson et al. (Ann Appl Probab 22(5):1989–2047, 2012) locates the critical size of *S*, above which it is likely that the infection will spread almost everywhere. Below this threshold, a central limit theorem is proved for the size of the eventually infected set. In this work, we calculate the rate function for the event that a small set *S* eventually infects an unexpected number of vertices, and identify the least-cost trajectory realizing such a large deviation.

## Introduction

Bootstrap percolation was originally proposed by physicists [12, 29] to model the phase transition observed in disordered magnets. Since then a large literature has developed, motivated by beautiful results, e.g. [8, 10, 22, 31], and a variety of applications across many fields, see e.g. [1, 2] and references therein.

In this work, we consider the spread of an infection by the *r*-neighbor bootstrap percolation dynamics on the Erdős–Rényi [15] graph ${\mathscr {G}}_{n,p}$, in which any two vertices in [*n*] are neighbors independently with probability *p*. Although we focus on this special case, we think our methods could be useful in studying the large deviations of any Markovian growth or exploration process. For instance, we have more recently used these methods to study the performance of the greedy independent set algorithm on sparse random graphs [26].

In bootstrap percolation, some subset $S_0\subset [n]$ is initially infected. Other vertices are infected once at least *r* of their neighbors become infected. Most of the literature has focused on the typical behavior. Of particular interest is the critical size at which point a uniformly random initial set $S_0$ is likely to infect most of the graph. Less is known about the atypical behavior, such as when a small set $S_0$ is capable of eventually infecting many more vertices than expected (e.g. influencers or superspreaders in a social network, viral marketing, etc.).

For analytical convenience, we rephrase the dynamics in terms of an exploration process (cf. [23, 30, 32]) in which vertices are infected one at a time. At any given step, vertices are either *susceptible,* *infected* or *healthy*. All susceptible vertices become infected eventually, and then remain infected. When a vertex is infected, some of the currently healthy vertices may become susceptible. The process ends once a stable configuration has been reached in which no vertices are susceptible.

More formally, at each step *t*, there are sets $I_t$ and $S_t$ of infected and susceptible vertices. Vertices in $[n]\setminus (I_t\cup S_t)$ are currently healthy. Initially, $I_0=\emptyset$. In step $t\ge 1$, some vertex $v_t\in S_{t-1}$ is infected. All remaining edges from $v_t$ are revealed. To obtain $S_t$ from $S_{t-1}$, we remove $v_t$ and add all neighbors of $v_t$ with exactly $r-1$ neighbors in $I_{t-1}$. We then add $v_t$ to $I_{t-1}$ to obtain $I_t$. The process ends at step $t_*=\min \{t\ge 1:S_t=\emptyset \}$ when no further vertices can be infected. For technical convenience, we set $|S_t|=0$ for all $t\ge t_*$. Let $I_*=I_{t_*}$ denote the eventually infected set. Since one vertex is infected in each step $t\le t_*$, we have $|I_t|=t$ and $|S_t|\ge |S_{t-1}|-1$ for all such *t*. In particular, $t_*=|I_*|$. Clearly, $I_*$ does not depend on the order in which vertices are infected.

Janson et al. [23] (cf. [34]) identifies the critical size of $S_0$, for all $r\ge 2$ and

1 $$\begin{aligned} p=((r-1)!/n)^{1/r}\vartheta ^{1/r-1}, \quad 1\ll \vartheta (n)\ll n, \end{aligned}$$

in the case that $S_0$ is selected uniformly at random. By the symmetry of ${\mathscr {G}}_{n,p}$, this is the same as for a given set $S_0$ (independent of ${\mathscr {G}}_{n,p}$) of the same size. More specifically, a sharp threshold is observed. If more than $(1-1/r)\vartheta$ vertices are initially susceptible, then all except *o*(*n*) many vertices are eventually infected. Otherwise, the eventually infected set is much smaller, of size $O(\vartheta )\ll n$.

### Theorem 1

([23] Theorem 3.1) Let *p* be as in (1) and $\alpha \ge 0$. Put $\alpha _r=(1-1/r)\alpha$. Suppose that a set $S_0=S_0(n)$ (independent of ${\mathscr {G}}_{n,p}$) of size $|S_0|\sim \alpha _r\vartheta$ is initially susceptible. If $\alpha >1$, then with high probability $|I_*|\sim n$. If $\alpha <1$, then with high probability $|I_*|\sim \varphi _\alpha \vartheta$, where $\varphi _\alpha \in [\alpha _r,\alpha ]$ uniquely satisfies

2 $$\begin{aligned} \varphi _\alpha -\varphi _\alpha ^r/r=\alpha _r. \end{aligned}$$

The extreme cases $p\sim c/n$ and $p\sim c/n^{1/r}$ are also addressed in [23], where the model behaves differently. We assume (1) throughout this work.

Moreover, in the subcritical case, a central limit theorem is proved in [23] (see Theorem 3.8). In this work, we study large deviations from the typical behavior in the subcritical case $\alpha <1$.

**Fig. 1:**
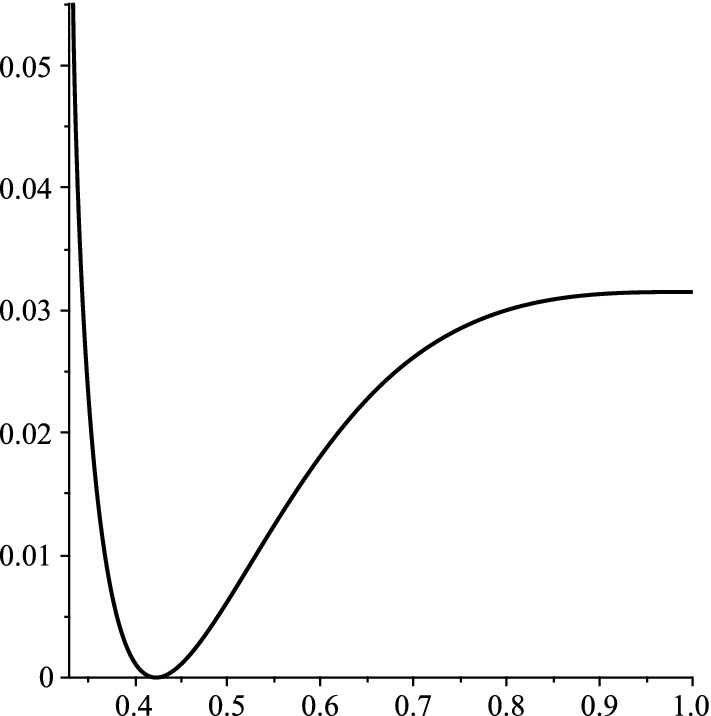
For $r=2$ and $\alpha =2/3$, the rate function $-\xi (\alpha ,\beta )$ is plotted as a function of $\beta$

### Definition 2

For $\beta < \varphi _\alpha$ (resp. $\beta > \varphi _\alpha$), let $P(S_0,\beta )$ denote the tail probability that the initial susceptibility of $S_0\subset [n]$ in ${\mathscr {G}}_{n,p}$ results in some number $|I_*|\le \beta \vartheta$ (resp. $|I_*|\ge \beta \vartheta$) of eventually infected vertices.

Informally, $P(S_0,\beta )$ is the probability that the number $|I_*|$ of eventually infected vertices is at least as atypical as $\beta \vartheta$.

### Theorem 3

Let *p* be as in (1), $\alpha \in [0,1)$ and $\beta \ne \varphi _\alpha \in [\alpha _r,1]$. Suppose that a set $S_0=S_0(n)$ (independent of ${\mathscr {G}}_{n,p}$) of size $|S_0|\sim \alpha _r\vartheta$ is initially susceptible. Then

$$\begin{aligned} \lim _{n\rightarrow \infty }\frac{1}{\vartheta }\log P(S_0,\beta ) =\xi (\alpha ,\beta ), \end{aligned}$$

where

3 $$\begin{aligned} \xi (\alpha ,\beta )= -\beta ^r/r+ {\left\{ \begin{array}{ll} (\beta -\alpha _r)[1+\log (\beta ^r/(r (\beta -\alpha _r))]&{}\beta \le \alpha \\ \alpha /r -(r-2)(\beta -\alpha ) +(r-1)\log (\beta ^\beta /\alpha ^{\alpha _r})&{}\beta >\alpha . \end{array}\right. } \end{aligned}$$

For any given $\alpha \in [0,1)$, $\xi (\alpha ,\beta )$ is increasing in $\beta \in [\alpha _r,\varphi _\alpha )$, decreasing in $\beta \in (\varphi _\alpha ,1]$ (see Appendix A.2), and $\xi (\alpha ,\varphi _\alpha )=0$ by (2), in line with Theorem 1. See Fig. 1.

**Fig. 2:**
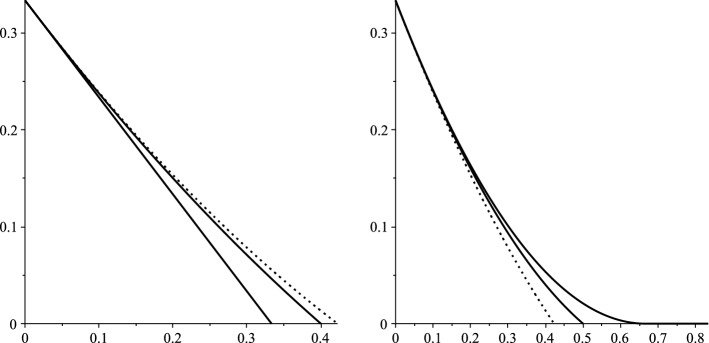
In both figures, $r=2$ and $\alpha =2/3$. The typical, zero-cost trajectory appears as a dotted line. Least-cost, deviating trajectories $\hat{y}_{\alpha ,\beta }$ for $\beta =1/3,2/5$ appear at left and $\beta =1/2,2/3,5/6$ at right

The asymptotically optimal trajectory $\hat{y}_{\alpha ,\beta }(x)$ for $|S_{x\vartheta }|/\vartheta$ is given at (9) below (see also Fig. 2). The rate function $\xi (\alpha ,\beta )$ is found by substituting this into the associated cost function (8). Detailed heuristics are given in Sect. 1.5 below. See Sect. 2 for the proof of Theorem 3.

The point $\vartheta$ (associated with $\beta =1$) is critical. As such, we simply have that $\xi (\alpha ,\beta )=\xi (\alpha ,1)$ for $\beta >1$. The reason for this is that the underlying branching process (the Binomial chain $|S_t|$ discussed in Sects. 1.4 and 1.5 below) governing the dynamics becomes critical upon surviving to time $t=\vartheta$. Surviving beyond this point, supposing that it has been reached, is no longer exponentially unlikely. In other words, the optimal (asymptotic) trajectory $\hat{y}(x)$ that $|S_{x\vartheta }|/\vartheta$ typically follows in order to survive beyond $x=1$ is equal to $\hat{y}_{\alpha ,1}(x)$ on [0, 1] (this has cost $-\xi (\alpha ,1)$). From then on ($x>1$), there is a zero-cost path that $\hat{y}(x)$ can follow.

We note here that in [23] (see Theorem 3.1) it is shown that $|I_*|/\vartheta$ converges to the typical value $\varphi _\alpha$ in probability. By Theorem 3 (and the Borel–Cantelli lemma) it follows that this convergence holds almost surely.

### Related Work

Torrisi et al. [33] established a full large deviations principle in the supercritical case, $\alpha >1$, where typically $|I_*|\sim n$. As discussed in [33], the main step in this regard is establishing sharp tail estimates (as in our Theorem 3 above). The full large deviations principle then follows by “elementary topological considerations.” Although we have not pursued it, we suspect that a full large deviations principle also holds in the present subcritical setting.

In closing, let us remark that it might be interesting to investigate the nature of ${\mathscr {G}}_{n,p}$, conditioned on the event that a given $S_0$ eventually infects a certain number of vertices, or on the existence of such a set $S_0$.

### Motivation

We came to this problem in studying *H-bootstrap percolation* on ${\mathscr {G}}_{n,p}$, as introduced by Balogh et al. [11], where all edges in ${\mathscr {G}}_{n,p}$ are initially infected and any other edge in an otherwise infected copy of *H* becomes infected. In the case that $H=K_4$, there is a useful connection with the usual *r*-neighbor bootstrap percolation model when $r=2$. Theorem 3 (when $r=2$ and $\vartheta =\Theta (\log n)$) plays a role (together with [9, 27]) in locating the critical probability $p_c\sim 1/\sqrt{3n\log n}$, where it becomes likely that all edges in $K_n$ are infected eventually. This solves an open problem in [11].

### Contagious Sets

A susceptible set $S_0$ is called *contagious* if it infects all of ${\mathscr {G}}_{n,p}$ eventually (i.e., $I_*=[n]$). Such sets have been studied for various graphs (e.g. [13, 18, 19, 28]). Recently, Feige et al. [16] considered the ${\mathscr {G}}_{n,p}$ case.

By Theorem 1, ${\mathscr {G}}_{n,p}$ has contagious sets of size $\Theta (\vartheta )$, however, there exist contagious sets that are much smaller. In [16], upper and lower bounds are obtained for the minimal size $m({\mathscr {G}}_{n,p},r)$ of a contagious set in ${\mathscr {G}}_{n,p}$. More recently [9], we showed that

4 $$\begin{aligned} p_c\sim [(r-1)!/n]^{1/r}[(\log {n})/(1-1/r)^2]^{1/r-1} \end{aligned}$$

is the sharp threshold for contagious sets of the smallest possible size *r*.

For $p<p_c$, Theorem 3 yields lower bounds for $m({\mathscr {G}}_{n,p},r)$ that sharpen those in [16] by a linear, multiplicative factor in *r*. Of course, *finding* sets of this size (if they exist) is a difficult and interesting problem (cf. the NP-complete problem of target set selection from viral marketing [14, 25]).

#### Corollary 4

Suppose that, for some $1\ll \vartheta \ll n$,

$$\begin{aligned} p=[(r-1)!/n]^{1/r}[\vartheta /(1-1/r)^2]^{1/r-1}. \end{aligned}$$

Then, with high probability,

$$\begin{aligned} m({\mathscr {G}}_{n,p},r) \ge (1-o(1)) r\vartheta /\log (n/\vartheta ). \end{aligned}$$

This result follows by an easy union bound, applying Theorem 3 in the case that $\alpha =0$ and $\beta =1$, see Appendix A.4.

By [9] this lower bound is sharp for *p* close to $p_c$, that is, when $\vartheta \sim \log n$. The methods in [9] might establish sharpness at least for $\vartheta \le O(\log {n})$.

### Binomial Chain

As in [23], we study the bootstrap percolation dynamics using the Binomial chain construction based on the work of Scalia-Tomba [30] (cf. Selke [32]). We only state here in this section the properties of this framework that we require, and refer the reader to Sect. 2 of [23] for the details.

Let $N_t$ be the number of vertices that have become susceptible during some time $s\in (0,t]$, so that $|S_t|=N_t-t+|S_0|$. By revealing edges (incident to infected vertices) on a need-to-know basis, the process $N_t$ can be expressed as the sum of $n-|S_0|$ independent and identically distributed processes, each of which is 0 until some $\mathrm{NegBin}(r,p)$ time, and then jumps to 1 (and remains at 1 thereafter). Informally, when a vertex is infected, it gives all of its neighbors a “mark.” A vertex, which was not initially susceptible, is susceptible or infected at a given time if it has received at least *r* marks by this time. In this way (see [23, 30]), it can be shown that $|S_t|$ is a Markov process, with

5 $$\begin{aligned} |S_t|\sim \mathrm{Bin}(n-|S_0|,\pi _t)-t+|S_0| \end{aligned}$$

where $\pi _t=\mathbf{P}(\mathrm{Bin}(t,p)\ge r)$. Moreover, its increments are distributed as

6 $$\begin{aligned} |S_t|-|S_s|\sim \mathrm{Bin}(n-|S_0|,\pi _t-\pi _s)-(t-s). \end{aligned}$$

### Heuristics

We first briefly recall the heuristic for Theorem 1 given in Sect. 6 of [23]. By the law of large numbers, with high probability $|S_t|\approx \mathbf{E}|S_t|$. A calculation shows that if $|S_0| > (1-1/r)\vartheta$ then $\mathbf{E}|S_t| > t$ for $t < n-o(n)$. On the other hand, if $|S_0|\sim \alpha _r\vartheta$, for some $\alpha <1$, then we have $\mathbf{E}|S_{\varphi _\alpha \vartheta }| \approx 0$. To see this, note that $pt=O[(\vartheta /n)^{1/r}]\ll 1$ for $t\le O(\vartheta )$ since $\vartheta \ll n$. Hence (see e.g. Sect. 8 of [23]) we have

$$\begin{aligned} \pi _t=\frac{(pt)^r}{r!}[1+O(pt+1/t)] \end{aligned}$$

and so

7 $$\begin{aligned} \mathbf{E}|S_{x\vartheta }|/\vartheta \sim x^r/r-x+\alpha _r. \end{aligned}$$

Next, we describe a natural heuristic, using the Euler–Lagrange equation, that allows us to anticipate the least-cost, deviating trajectories (the functions $\hat{y}_{\alpha ,\beta }$ in (9) below), which lead to Theorem 3. The proof, given in Sect. 2 below, makes this rigorous by a discrete analogue of the Euler–Lagrange equation. We think this method will be of use in studying the tail behavior of other random processes.

Consider a trajectory $y(x)\ge 0$ from $\alpha _r$ to 0 over $[0,\beta ]$. Suppose that $|S_{x\vartheta }|/\vartheta$ has followed this trajectory until step $t-1=x\vartheta$. In the next step *t*, some vertex $v_t\in S_{t-1}$ is infected. There are approximately a Poisson with mean $np^r{t-1\atopwithdelims ()r-1}\approx x^{r-1}$ (this approximation holds by (1) and standard combinatorial estimates) number of vertices that are neighbors with $v_t$ and $r-1$ of the $t-1$ vertices infected in previous steps $s<t$. Such vertices become susceptible in step *t*. Therefore, to continue along this trajectory, we require this Poisson random variable to take the value

$$\begin{aligned} 1+\vartheta [y(x+1/\vartheta )-y(x)]\approx 1+y'(x). \end{aligned}$$

(The “$+1$” accounts for the vertex $v_t\in S_{t-1}$ that is infected in step *t*, and so removed from the next susceptible set $S_t$.) As is well-known, this event has approximate log probability $-\Gamma ^*_{x^{r-1}}(1+y'(x))$, where

$$\begin{aligned} \Gamma ^*_{\lambda }(u)=-u[1-\lambda /u+\log (\lambda /u)] \end{aligned}$$

is the Legendre–Fenchel transformation of the cumulant-generating function of a mean $\lambda$ Poisson. Hence $|S_{x\vartheta }|/\vartheta \approx y(x)$ on $[0,\beta ]$ with approximate log probability

8 $$\begin{aligned} \vartheta \int _0^\beta (1+y'(x))\left[ 1-\frac{x^{r-1}}{1+y'(x)}+\log \frac{x^{r-1}}{1+y'(x)}\right] dx \end{aligned}$$

(cf. (13) below). Maximizing this integral is particularly simple, since the integrand depends on $y'$, but not *y*. The Euler–Lagrange equation implies that the least-cost trajectory satisfies

$$\begin{aligned} \frac{d}{dx}\log \frac{x^{r-1}}{1+y'(x)}=0 \implies y(x)=(\beta -\alpha _r)(x/\beta )^r-x+\alpha _r, \end{aligned}$$

except where possibly the boundary constraint $y(x)\ge 0$ might intervene.

Since, as noted above, $|S_t|\ge |S_{t-1}|-1$ for all *t*, we may assume that $\beta \ge \alpha _r$. That is, any trajectory *y*(*x*) of $|S_{x\vartheta }|/\vartheta$ decreases no faster than $-x$. Also note that $(\alpha -\alpha _r)/\alpha ^r=1/(r\alpha ^{r-1})$, and that for any larger $b>1/(r\alpha ^{r-1})$ the function $bx^r-x+\alpha _r$ has no zeros in [0, 1].

As it turns out, the least-cost trajectory from $\alpha _r$ to 0 over $[0,\beta ]$ is9where $\alpha \wedge \beta =\min \{\alpha ,\beta \}$. Setting $\beta =\varphi _\alpha$, we recover by (2) the typical, zero-cost trajectory (7). See Fig. 2. Substituting (9) into (8), we obtain $\vartheta \xi (\alpha ,\beta )$ after some basic calculus (see Appendix A.1 below).

## Proof of Theorem 3

Before turning to the proof, let us recall Theorem 3 and the definitions involved. We fix some $\alpha \in [0,1)$ and $\beta \ne \varphi _\alpha \in [\alpha _r,1]$ (with $\varphi _\alpha$ as defined at (2)). We assume that $|S_0|/\vartheta \rightarrow \alpha _r=(1-1/r)\alpha$ as $n\rightarrow \infty$, where $S_0=S_0(n)$ is the initially susceptible set. Recall that $I_*=I_{t_*}$ is the eventually infected set, where $t_*$ is the first time *t* that $|S_t|=0$ (no susceptible vertices). Finally, recall that Theorem 3 identifies the limit of $(1/\vartheta )\log P(S_0,\beta )$, where $P(S_0,\beta )$ is the tail probability that $|I_*|\le \beta \vartheta$ if $\beta <\varphi _\alpha$ or $|I_*|\ge \beta \vartheta$ if $\beta >\varphi _\alpha$.

Upper bounds for $P(S_0,\beta )$ are established in Sect. 2.1 ($\beta <\varphi _\alpha$) and Sect. 2.2 ($\beta >\varphi _\alpha$) below. The main idea is to use a discrete version of the Euler–Lagrange equation to identify the asymptotically optimal trajectory $\hat{y}(x)$ of the process $|S_{x\vartheta }|/\vartheta$ realizing the associated event ($|I_*|\le \beta \vartheta$ or $|I_*|\ge \beta \vartheta$ depending on $\beta$). It turns out that $\hat{y} = \hat{y}_{\alpha ,\beta }$ (see (9) above). More specifically, we use the following result, which is a special case of Theorem 5 in Guseinov [20]. See also [3–7, 17, 24] and references therein for earlier related results and background.

Let $\Delta x_i=x_{i+1}-x_i$ denote the forward difference operator.

### Lemma 5

Fix $a,b\in {\mathbb {R}}$, a function *f*(*u*, *v*) with continuous partial derivatives $f_u$ and $f_v$, and evenly spaced points $x_0\le x_1\le \cdots \le x_m$. Then the maximizer $\hat{y}$ of

$$\begin{aligned} \sum _{i=0}^{m-1} f(x_{i+1},\Delta y_i/\Delta x_i)\Delta x_i, \end{aligned}$$

over trajectories with $y_0=a$ and $y_m=b$, satisfies $f_v(x_{i+1},\Delta \hat{y}_i/\Delta x_i)\equiv c$ for some constant *c*.

The proof of this result amounts to adding a Lagrange multiplier to constrain $\sum _i\Delta y_i$ and then comparing the derivative to 0. A more general version, more closely resembling the regular Euler–Lagrange equation, appears in [20]. This allows for more complicated functions $f(x_i,x_{i+1},y_i,y_{i+1},\Delta y_i/\Delta x_i)$ and points $x_i$ that need not be evenly spaced. The proof is analogous to that of its continuous counterpart, using summation by parts instead of integration by parts, for instance.

Finally, in Sect. 2.3, we establish asymptotically equivalent lower bounds for $P(S_0,\beta )$ by considering specific trajectories *y* that are asymptotically equivalent to $\hat{y}_{\alpha ,\beta }$. This altogether verifies the asymptotic optimality of $\hat{y}_{\alpha ,\beta }$ and the convergence of $(1/\vartheta )\log P(S_0,\beta )$.

### Upper Bounds When $\beta <\varphi _\alpha$

We begin with the simpler case that $\beta <\varphi _\alpha$. The opposite case $\beta >\varphi _\alpha$ follows by an elaboration of these arguments (see Sect. 2.2 below). Since $\beta <\varphi _\alpha$, note that $P(S_0,\beta )$ is simply the probability that $|S_{x\vartheta }|=0$ for some $x\le \beta$, as this occurs if and only if $|I_*|\le \beta \vartheta$.

To begin, we discretize the unit interval [0, 1] as follows. Let $m=\lceil \vartheta /(\log \vartheta )^2\rceil$. Consider the points $x_i = (i/\vartheta )\lceil (\log \vartheta )^2 \rceil$, for $i=0,1,\ldots ,m$. Note that the points $x_i\vartheta$ are evenly spaced integers. Also note that $x_m\sim 1$, since $\vartheta \gg 1$.

Let ${\mathscr {Y}}_{n}$ denote the set of trajectories $y_i=|S_{x_i\vartheta }|/\vartheta$ such that

1. all $$y_i \vartheta \in {\mathbb {Z}}$$,
2. $$y_0\vartheta =|S_0|$$,
3. all $$\Delta y_i/\Delta x_i\ge -1$$, and
4. $$y_i=0$$ for all $$x_i\ge \beta $$.

Note that we can assume (3) since, as discussed above, $|S_t|\ge |S_{t-1}|-1$ for all *t*. Since $|S_t|$ is Markov,

$$\begin{aligned} P(S_0,\beta )\le \sum _{y\in {\mathscr {Y}}_{n}}\prod _{i=0}^{m-1} \mathbf{P}\left( \frac{|S_{x_{i+1}\vartheta }|}{\vartheta }= y_{i+1} | \frac{|S_{x_{i}\vartheta }|}{\vartheta }= y_{i}\right) . \end{aligned}$$

By (3) and (4) it follows that all $y_i\le \beta$ for any $y\in {\mathscr {Y}}_{n}$. Hence $|{\mathscr {Y}}_{n}|\le \vartheta ^m$. Therefore, taking a union bound,

$$\begin{aligned} P(S_0,\beta )\le \vartheta ^m \prod _{i=0}^{m-1} \mathbf{P}\left( \frac{|S_{x_{i+1}\vartheta }|}{\vartheta }= \hat{y}_{i+1} | \frac{|S_{x_{i}\vartheta }|}{\vartheta }= \hat{y}_{i}\right) , \end{aligned}$$

where $\hat{y}$ maximizes the product over $y\in {\mathscr {Y}}_{n}$. Noting that $(m/\vartheta )\log \vartheta \ll 1$, we find altogether that

10 $$\begin{aligned} \frac{1}{\vartheta }\log P(S_0,\beta ) \le o(1) +\frac{1}{\vartheta }\sum _{i=0}^{m-1} \log \mathbf{P}\left( \frac{|S_{x_{i+1}\vartheta }|}{\vartheta }=\hat{y}_{i+1} | \frac{|S_{x_{i}\vartheta }|}{\vartheta }=\hat{y}_{i}\right) . \end{aligned}$$

We now turn to the issue of identifying $\hat{y}\in {\mathscr {Y}}_{n}$. By (5) it follows that

11 $$\begin{aligned} \Delta |S_{x_i\vartheta }|\sim \mathrm{Bin}(n-|S_0|,\Delta \pi (x_i\vartheta ))-\vartheta \Delta x_i. \end{aligned}$$

Hence, using the standard bound ${n\atopwithdelims ()k}\le (en/k)^k$ and $1-x\le e^{-x}$,

12 $$\begin{aligned}&\mathbf{P}\left( \frac{|S_{x_{i+1}\vartheta }|}{\vartheta }=y_{i+1} | \frac{|S_{x_{i}\vartheta }|}{\vartheta }=y_{i}\right) \nonumber \\&\quad = \mathbf{P}(\mathrm{Bin}(n-|S_0|,\Delta \pi (x_i\vartheta ))=\vartheta (\Delta x_i+\Delta y_i)\nonumber \\&\quad \le \left( e\frac{n\Delta \pi (x_i\vartheta )}{\vartheta (\Delta x_i+\Delta y_i)}\right) ^{\vartheta (\Delta x_i+\Delta y_i)} [1-\Delta \pi (x_i\vartheta )]^{n-|S_0|-\vartheta (\Delta x_i+\Delta y_i)}\nonumber \\&\quad \le \left( e\frac{n\Delta \pi (x_i\vartheta )}{\vartheta (\Delta x_i+\Delta y_i)}\right) ^{\vartheta (\Delta x_i+\Delta y_i)} e^{-n\Delta \pi (x_i\vartheta )}\nonumber \\&\quad \times e^{(|S_0|+\vartheta (\Delta x_i+\Delta y_i))\Delta \pi (x_i\vartheta )}. \end{aligned}$$

(We have written the upper bound in this way so as to compare with the lower bound at (18) below.)

Before substituting this upper bound into (10), we collect the following technical result. The proof is elementary, though somewhat tedious, see Sect. Appendix A.5 below. Note that by (1), $1\ll \vartheta \ll 1/p$.

#### Lemma 6

We have that

$$\begin{aligned} \frac{rn}{\vartheta }\frac{\Delta \pi (x_i\vartheta )}{\Delta (x_i^r)} =1+O\left( p\vartheta +\frac{1}{\log \vartheta }\right) \sim 1. \end{aligned}$$

Altogether, we find that

$$\begin{aligned}&\frac{1}{\vartheta }\sum _{i=0}^{m-1} \log \mathbf{P}\left( \frac{|S_{x_{i+1}\vartheta }|}{\vartheta }= y_{i+1} | \frac{|S_{x_{i}\vartheta }|}{\vartheta }= y_{i}\right) \\&\quad \le o(1)+\sum _{i=0}^m(\Delta x_i+\Delta y_i)\left[ 1-\frac{\Delta (x_i^r)/r}{\Delta x_i+\Delta y_i}+\log \frac{\Delta (x_i^r)/r}{\Delta x_i+\Delta y_i}\right] . \end{aligned}$$

Since $\log x-x$ is increasing for $x\in (0,1]$ and $\Delta (x_i^r)/r\le x_{i+1}^{r-1}\Delta x_i$, it follows by (10) that

13 $$\begin{aligned} \frac{1}{\vartheta }\log P(S_0,\beta ) \le o(1)+\sum _{i=0}^{m-1}f(x_{i+1},\Delta \hat{y}_i/\Delta x_i)\Delta x_i, \end{aligned}$$

where

14 $$\begin{aligned} f(u,v)=(1+v)\left[ 1-\frac{u^{r-1}}{1+v}+\log \frac{u^{r-1}}{1+v}\right] \end{aligned}$$

(cf. (8) above) and $\hat{y}\in {\mathscr {Y}}_n$ maximizes the sum in (13).

In order to apply Lemma 5, we lift the restriction that all $y_i\vartheta \in {\mathbb {Z}}$, and maximize

$$\begin{aligned} \sum _{i=0}^{m-1}f(x_{i+1},\Delta y_i/\Delta x_i)\Delta x_i \end{aligned}$$

over $y\in {\mathbb {R}}^{m+1}$ with (i) $y_0=\alpha _r$, (ii) $\Delta y_i/\Delta x_i\ge -1$ and (iii) $y_i=0$ for all $x_i\ge \beta$. By Lemma 5, the maximizer $\hat{y}=\hat{y}(n)$ satisfies

$$\begin{aligned} \Delta f_v(x_{i+1},\Delta \hat{y}_i/\Delta x_i) \equiv 0 \end{aligned}$$

between any two given points where $\hat{y}>0$. Since

$$\begin{aligned} f_v(u,v)=\log \frac{u^{r-1}}{1+v} \end{aligned}$$

this implies that $1+\Delta \hat{y}_i/\Delta x_i=bx_{i+1}^{r-1}$, for some constant *b*, between any two points $x_j<x_k$ where $\hat{y}_i>0$ for $j<i<k$. On the other hand, if both $\hat{y}_j=\hat{y}_k=0$, then necessarily $\hat{y}_i=0$ for $j<i<k$. By standard results on the Euler approximation of differential equations (see e.g. Theorems 7.3 and 7.5 in Sect. I.7 of [21]), it follows that, on all segments where $\hat{y}_i>0$, the discrete derivative $\Delta \hat{y}_i/\Delta x_i$ is within *O*(1/*m*) of the function $bx^{r-1}-1$, for some $b=b(n)$.

Altogether, in the limit, it suffices to consider trajectories that take the form $(\beta '-\alpha _r)(x/\beta ')^r-x+\alpha _r$ (until they hit 0), for some $\beta '\in [\alpha _r,\beta ]$, since (as discussed in Sect. 1.5) these are the only functions $y(x)=bx^r-x+\alpha _r$ for which (i) $y(0)=\alpha _r$, (ii) $y'(x)\ge -1$ and (iii) $y(x)=0$ for some $x\le \beta$. Hence, by the above considerations, and the continuity of *f*, we find that

15 $$\begin{aligned} \limsup _{n\rightarrow \infty } \frac{1}{\vartheta }\log P(S_0,\beta ) \le \sup _{\beta '\in [\alpha _r,\beta ]}\int _0^{\beta '}f(x,\hat{y}'_{\alpha ,\beta '}(x))dx. \end{aligned}$$

To conclude, we observe, by Appendices A.1 and A.2, that the right hand side equates to

$$\begin{aligned} \sup _{\beta '\in [\alpha _r,\beta ]}\xi (\alpha ,\beta ')=\xi (\alpha ,\beta ). \end{aligned}$$

### Upper bounds When $\beta >\varphi _\alpha$

The case $\beta >\varphi _\alpha$ follows by the same method of proof, however, there are two additional technical complications. Specifically, (i) the set of relevant trajectories ${\mathscr {Y}}_{n}$ in this case (defined below) no longer satisfies $|{\mathscr {Y}}_{n}|\le [O(\vartheta )]^m$, and (ii) to obtain an upper bound for $(1/\vartheta )\log P(S_0,\beta )$, as in (15) above, we need to take a supremum over a more complicated set of trajectories. This latter issue is due in part to the fact that is not a priori clear that the optimal trajectory $\hat{y}$ should hit 0 before $x=1$ (that is, that $\hat{y}$ is one of $\hat{y}_{\alpha ,\beta }$). This indeed turns out to be the case, however, even so, $\hat{y}_{\alpha ,\beta }$ is slightly more complicated (defined piecewise) when $\beta >\alpha$.

First note that, for $\beta >\varphi _\alpha$, $P(S_0,\beta )$ is the probability that $|S_{x\vartheta }|>0$ for all $x< \beta$. Therefore, in this case, we take ${\mathscr {Y}}_{n}$ to be the set of $y_i=|S_{x_i\vartheta }|/\vartheta$ for which

1. all $$y_i \vartheta \in {\mathbb {Z}}$$,
2. $$y_0\vartheta =|S_0|$$,
3. all $$\Delta y_i/\Delta x_i\ge -1$$, and
4. $$y_i>0$$ for all $$x_i<\beta $$.

We no longer have that $|{\mathscr {Y}}_{n}|\le [O(\vartheta )]^m$. However, for $t\le O(\vartheta )$, by (5) and Chernoff’s bound,

$$\begin{aligned} \frac{1}{\vartheta }\log \mathbf{P}( |S_{t}| \ge (1+\delta )\vartheta ) \le -O(\delta ^2). \end{aligned}$$

Therefore, for *A* sufficiently large, the log probability that any $|S_t|>A\vartheta$ while $t\le O(\vartheta )$ is less than $\vartheta \xi (\alpha ,\beta )$. Hence, arguing as the previous section, we find that

16 $$\begin{aligned} \limsup _{n\rightarrow \infty } \frac{1}{\vartheta }\log P(S_0,\beta ) \le \sup _{y\in {\mathscr {Y}}}\int f(x,y'(x))dx, \end{aligned}$$

where ${\mathscr {Y}}$ is the set of non-negative trajectories *y*(*x*) that start at $y(0)=\alpha _r$ and take the form $bx^r-x+a$, for some $b\ge 0$, wherever they are positive. However, it suffices to consider a smaller set than ${\mathscr {Y}}$. Indeed, observe that the maximizer $\hat{y}\in {\mathscr {Y}}$ is non-increasing. This is intuitive, since the process is sub-critical while the total number of infected vertices remains less than $\vartheta$. To see this formally, note that (i) the derivative of any trajectory $bx^r-x+a$ is $brx^{r-1}-1\le 0$ for any $x\le 1$ unless $b>1/r$, and (ii) we have by (14) that

$$\begin{aligned} f(x,brx^{r-1}-1)=[br-1-br\log (br)]x^{r-1} \end{aligned}$$

is decreasing in $b>1/r$. Hence, it suffices to consider trajectories which take the form $(\beta '-\alpha _r)(x/\beta ')^r-x+\alpha _r$ until they hit 0 at some $\beta '\in [\alpha _r,\alpha ]$, and then, if $\beta '<\beta$, are 0 thereafter until $x=\beta$. (Note that, for any $b>1/(r\alpha ^{r-1})$, the function $bx^r-x+\alpha _r$ has no zeros and, since $\alpha <1$, is increasing eventually on [0, 1].) Therefore, by Appendix A.1,

$$\begin{aligned} \limsup _{n\rightarrow \infty } \frac{1}{\vartheta }\log P(S_0,\beta )&\le \sup _{\beta '\in [\alpha _r,\alpha ]} \left[ \int _0^{\beta '} f(x, y'_{\alpha ,\beta '}(x))dx+\mathbf{1}_{\beta '<\beta }\int _{\beta '}^\beta f(x,0)dx\right] \\&=\sup _{\beta '\in [\alpha _r,\alpha ]} \left[ \xi (\alpha ,\beta ')+\mathbf{1}_{\beta '<\beta }\int _{\beta '}^\beta f(x,0)dx\right] \end{aligned}$$

By basic calculus (see Appendix A.3) it can be shown that the right hand side is bounded by $\xi (\alpha ,\beta )$.

### Lower Bounds

The lower bound is much simpler. As discussed above, it essentially suffices to consider any trajectory $y(x)\sim \hat{y}_{\alpha ,\beta }(x)$ which contributes to $P(S_0,\beta )$, and show that the scaled log probability that $|S_{x\vartheta }|/\vartheta$ follows this trajectory is asymptotic to $\xi (\alpha ,\beta )$.

Once again, there is some asymmetry in the cases $\beta <\varphi _\alpha$ and $\beta >\varphi _\alpha$ due to the definition of $P(S_0,\beta )$. For $\beta <\varphi _\alpha$, we note that if, for instance, all $|S_{x_i\vartheta }|/\vartheta =\tilde{y}_i$, where

$$\begin{aligned} \tilde{y}_i=\frac{1}{\vartheta }\lfloor \hat{y}_{\alpha ,\beta }(x_i)\vartheta \rfloor \mathbf{1}_{x_i\le \beta -\Delta x_i/\vartheta }, \end{aligned}$$

then $|I_*|\le \beta \vartheta$. The indicator present here ensures that $|S_{x\vartheta }|$ hits 0 by $x=\beta$. On the other hand, if $\beta >\varphi _\alpha$, set

$$\begin{aligned} \tilde{y}_i=\frac{1}{\vartheta }\lceil \hat{y}_{\alpha ,\beta }(x_i)\vartheta \rceil +\Delta x_i\mathbf{1}_{x_i<\beta }. \end{aligned}$$

Then if all $|S_{x_i\vartheta }|/\vartheta =\tilde{y}_i$ we have $|I_*|\ge \beta \vartheta$. The indicator in this case ensures that $|S_t|>0$ between increments while $t<\beta \vartheta$.

Next, we show that

17 $$\begin{aligned} \liminf _{n\rightarrow \infty } \frac{1}{\vartheta } \sum _{i=0}^{m-1} \log \mathbf{P}\left( \frac{|S_{x_{i+1}\vartheta }|}{\vartheta }= \tilde{y}_{i+1} | \frac{|S_{x_{i}\vartheta }|}{\vartheta }= \tilde{y}_{i}\right) \ge \xi (\alpha ,\beta ), \end{aligned}$$

since then, by Sect. 2.1 and 2.2, it follows that

$$\begin{aligned} \lim _{n\rightarrow \infty } \frac{1}{\vartheta }\log P(S_0,\beta )=\xi (\alpha ,\beta ), \end{aligned}$$

as stated in Theorem 3.

To this end, note that by (11) and the standard bounds ${n\atopwithdelims ()k}\ge (n-k)^k/k!$, $k!\le ek(k/e)^k$ and $(1-x)^n\ge e^{-xn}(1-nx^2)$, it follows that

18 $$\begin{aligned}&\mathbf{P}\left( \frac{|S_{x_{i+1}\vartheta }|}{\vartheta }=y_{i+1} | \frac{|S_{x_{i}\vartheta }|}{\vartheta }=y_{i}\right) \nonumber \\&\quad \ge \left( e\frac{n\Delta \pi (x_i\vartheta )}{\vartheta (\Delta x_i+\Delta y_i)}\right) ^{\vartheta (\Delta x_i+\Delta y_i)} e^{-n\Delta \pi (x_i\vartheta )}\nonumber \\&\quad \times \frac{(1-n(\Delta \pi (x_i\vartheta ))^2)}{e\vartheta (\Delta x_i+\Delta y_i)} \left( 1-\frac{|S_0|+\vartheta (\Delta x_i+\Delta y_i)}{n}\right) ^{\vartheta (\Delta x_i+\Delta y_i)} \end{aligned}$$

(cf. (12)). Therefore, in a similar way as for (13) above (however instead using $\Delta (x_i^r)/r\ge x_{i}^{r-1}\Delta x_i$), we find that

$$\begin{aligned} \frac{1}{\vartheta } \sum _{i=0}^{m-1} \log \mathbf{P}\left( \frac{|S_{x_{i+1}\vartheta }|}{\vartheta }= \tilde{y}_{i+1} | \frac{|S_{x_{i}\vartheta }|}{\vartheta }= \tilde{y}_{i}\right) \ge o(1)+\sum _{i=0}^{m-1}f(x_{i},\Delta \tilde{y}_i/\Delta x_i)\Delta x_i, \end{aligned}$$

where *f*, once again, is as defined at (14). Therefore, by the choice of $\tilde{y}_i$, it can be seen (using Appendix A.1) that

$$\begin{aligned} \liminf _{n\rightarrow \infty } \frac{1}{\vartheta } \sum _{i=0}^{m-1} \log \mathbf{P}\left( \frac{|S_{x_{i+1}\vartheta }|}{\vartheta }= \tilde{y}_{i+1} | \frac{|S_{x_{i}\vartheta }|}{\vartheta }= \tilde{y}_{i}\right) \ge \int _0^\beta f(x,\hat{y}_{\alpha ,\beta }'(x))=\xi (\alpha ,\beta ), \end{aligned}$$

yielding (17), and thus concluding the proof of Theorem 3.

## Appendix A: Technical Results

This section contains several technical results, all of which follow by elementary methods.

### A.1: Rate Function $\xi$

We show that

$$\begin{aligned} \int _0^\beta f(x,\hat{y}_{\alpha ,\beta }'(x))dx=\xi (\alpha ,\beta ), \end{aligned}$$

where $\hat{y}_{\alpha ,\beta }$ and *f* are as in (9) and (14) above (that is, the cost of the least-cost trajectory $\hat{y}_{\alpha ,\beta }(x)$ over $[0,\beta ]$ is $-\xi (\alpha ,\beta )$).

First note that, whenever $y_{\alpha ,\beta }(x)>0$,

$$\begin{aligned} 1+y'_{\alpha ,\beta }(x)=\frac{r(\beta -\alpha _r)}{\beta ^r} x^{r-1}, \end{aligned}$$

in which case

$$\begin{aligned} f(x,\hat{y}'_{\alpha ,\beta }(x))= rx^{r-1}\frac{(\beta -\alpha _r)}{\beta ^r}\left( 1-\frac{\beta ^r}{r(\beta -\alpha _r)}+\log \frac{\beta ^r}{r(\beta -\alpha _r)}\right) . \end{aligned}$$

Hence, if $$\beta \le \alpha $$,

$$\begin{aligned} \int _0^\beta f(x,\hat{y}_{\alpha ,\beta }'(x))dx =(\beta -\alpha _r)\left( 1-\frac{\beta ^r}{r(\beta -\alpha _r)}+\log \frac{\beta ^r}{r(\beta -\alpha _r)}\right) =\xi (\alpha ,\beta ). \end{aligned}$$

On the other hand, note that

$$\begin{aligned} f(x,0)=1-x^{r-1}+(r-1)\log x \end{aligned}$$

and so

$$\begin{aligned} \int f(x,0)dx=-x^r/r-(r-2)x+(r-1)x\log x. \end{aligned}$$

Therefore, if $\beta >\alpha$, then we find (after some algebraic simplifications) that

$$\begin{aligned}&\int _0^\beta f(x,\hat{y}_{\alpha ,\beta }'(x))dx =\xi (\alpha ,\alpha )+\int _\alpha ^\beta f(x,0)dx\\&\quad =\frac{\alpha -\alpha ^r}{r}+(r-1)\log (\alpha ^{\alpha /r}) -\frac{\beta ^r-\alpha ^r}{r}-(r-2)(\beta -\alpha ) +(r-1)\log \frac{\beta ^\beta }{\alpha ^\alpha }\\&\quad =-\frac{\beta ^r-\alpha }{r}-(r-2)(\beta -\alpha )+(r-1)\log \frac{\beta ^\beta }{\alpha ^{\alpha _r}} =\xi (\alpha ,\beta ). \end{aligned}$$

### A.2: Shape of $\xi$

We note here that $\xi (\alpha ,\beta )$ is increasing in $\beta \in [\alpha _r,\varphi _\alpha )$ and decreasing in $\beta \in (\varphi _\alpha ,1]$ (as in Fig. 1 above). When $\beta \le \alpha$,

$$\begin{aligned} \frac{\partial }{\partial \beta }\xi (\alpha ,\beta ) = \log \frac{\beta ^r/r}{\beta -\alpha _r}+r(1-\alpha _r/\beta )-\beta ^{r-1}. \end{aligned}$$

Therefore, if $\beta \in [\alpha _r,\varphi _\alpha ]$, by (2) and $\log x\ge 1-1/x$,

$$\begin{aligned} \frac{\partial }{\partial \beta }\xi (\alpha ,\beta ) \ge \frac{1-\beta ^{r-1}}{\beta ^r/r}(\alpha _r-\beta +\beta ^r/r)\ge 0. \end{aligned}$$

On the other hand, if $\beta \in [\varphi _\alpha ,\alpha ]$, by (2) and $\log x\le x-1$,

$$\begin{aligned} \frac{\partial }{\partial \beta }\xi (\alpha ,\beta ) \le \frac{(r-1)(\alpha -\beta )}{\beta (\beta -\alpha _r)}(\alpha _r-\beta +\beta ^r/r)\le 0. \end{aligned}$$

Finally, if $\beta \in [\alpha ,1]$, note that

$$\begin{aligned} \frac{\partial }{\partial \beta }\xi (\alpha ,\beta ) =1-\beta ^{r-1}+(r-1)\log \beta \le 1-\beta +\log \beta \le 0. \end{aligned}$$

### A.3: An Inequality Involving $\xi$

We show that, for any $\beta >\varphi _\alpha$ and $\beta '\in [\alpha _r,\alpha ]$,

$$\begin{aligned} \xi (\alpha ,\beta ')+\mathbf{1}_{\beta '<\beta }\int _{\beta '}^\beta f(x,0)dx \le \xi (\alpha ,\beta ). \end{aligned}$$

If $\beta '\ge \beta$ the result is immediate, since by Appendix A.2 we have $\xi (\alpha ,\beta ')\le \xi (\alpha ,\beta )$ in this case. Hence, assuming that $\beta <\beta '$, we show that

$$\begin{aligned} \int _{\beta '}^\beta f(x,0)dx \le \xi (\alpha ,\beta )-\xi (\alpha ,\beta '). \end{aligned}$$

If $$\beta >\alpha $$, then

$$\begin{aligned} \xi (\alpha ,\alpha )+\int _{\alpha }^\beta f(x,0)dx=\xi (\alpha ,\beta ), \end{aligned}$$

so we may further assume that $\beta \le \alpha$. As has already been noted above, $f(x,0)=1-x^{r-1}+(r-1)\log x$, and so

$$\begin{aligned} \int f(x,0)dx=-x^r/r-(r-2)x+(r-1)x\log x. \end{aligned}$$

Hence by (3) it suffices to show that

$$\begin{aligned} \xi (\alpha ,x)-\int f(x,0)dx= (r-1)(x-x\log x)+(x-\alpha _r)\log \frac{x^r}{r(x-\alpha _r)} \end{aligned}$$

is increasing in $x\ge \alpha _r$. Differentiating the above expression with respect to *x*, we obtain (after some straightforward simplifications)

$$\begin{aligned} \frac{r(x-\alpha _r)}{x}-\log \frac{r(x-\alpha _r)}{x}-1\ge 0, \end{aligned}$$

yielding the claim.

### A.4: Lower Bound for $m({\mathscr {G}}_{n,p},r)$

#### Proof of Theorem 4

For $\delta >0$, let $t_\delta =(1-\delta )r\vartheta /\log (n/\vartheta )$. We show that, with high probability, ${\mathscr {G}}_{n,p}$ has no contagious sets smaller than $t_\delta$. Note that

$$\begin{aligned} \xi (0,1)\vartheta /(1-1/r)^2=-r\vartheta . \end{aligned}$$

The expected number of subsets $S_0\subset [n]$ of size $|S_0|=t_\delta$ which if initially susceptible cause $|I_*|\ge \vartheta /(1-1/r)^2$ vertices to be infected eventually is at most

$$\begin{aligned} {n\atopwithdelims ()t_\delta } e^{-r\vartheta (1+o(1))} \le (ne/t_\delta )^{t_\delta } e^{-r\vartheta (1+o(1))} =e^{-r\vartheta \psi }, \end{aligned}$$

where

$$\begin{aligned} \psi = 1+o(1) -(1-\delta )\log (ne/t_\delta )/\log (n/\vartheta ). \end{aligned}$$

Since

$$\begin{aligned} \log (ne/t_\delta )\le \log (n/\vartheta ) +O\left( \log \log (n/\vartheta )\right) , \end{aligned}$$

$\psi >0$ for all large *n*, and the result follows. $\square$

### A.5: Increments of $\pi$

#### Proof of Lemma 6

Recall that

$$\begin{aligned} m=\Theta \left( \frac{1}{\Delta x_i}\right) =\Theta \left( \frac{\vartheta }{(\log \vartheta )^2}\right) . \end{aligned}$$

When $i=0$, we have $\Delta \pi (x_i\vartheta )=\pi (x_1\vartheta )$ since $x_0=0$. By the estimates discussed in Sect. 1.5,

$$\begin{aligned} \frac{rn}{\vartheta }\pi (x_1\vartheta ) = x_1^r\left[ 1+O\left( p\vartheta +\frac{1}{(\log \vartheta )^2}\right) \right] . \end{aligned}$$

Next, we assume that $i\ge 1$. Then $x_{i+1}\le O(x_i)$ and, for all $\ell \ge r$,

19 $$\begin{aligned} 1\le \frac{\Delta (x_i^\ell )}{\ell x_i^{\ell -1}\Delta x_i}\le O(1)^\ell . \end{aligned}$$

For the lower bound, first note that

$$\begin{aligned} \mathbf{P}(\mathrm{Bin}(x_{i+1}\vartheta ,p)>r)> \mathbf{P}(\mathrm{Bin}(x_{i}\vartheta ,p)>r) \end{aligned}$$

and so

$$\begin{aligned} \Delta \pi (x_i\vartheta ) > \Delta \mathbf{P}(\mathrm{Bin}(x_{i}\vartheta ,p)=r). \end{aligned}$$

Hence, using (1) and (19) (and the standard bounds $(n-k)^k\le {n\atopwithdelims ()k}k!\le n^k$ and $(1-x)^y\ge 1-xy$) we find

$$\begin{aligned} \frac{rn}{\vartheta }\Delta \pi (x_i\vartheta )&\ge (1-p)^{x_{i}\vartheta -r}\left[ \left( x_{i+1}-\frac{r}{\vartheta }\right) ^r (1-p)^{\vartheta \Delta x_{i}}- x_i^r\right] \\&\ge \Delta (x_{i}^r) (1-p\vartheta )\left[ 1- \frac{x_{i+1}^r}{\Delta (x_{i}^r)}\left( p\vartheta \Delta x_{i}+\frac{r^2}{x_{i+1}\vartheta }\right) \right] \\&= \Delta (x_{i}^r) \left[ 1- O\left( p\vartheta +\frac{1}{(\log \vartheta )^2}\right) \right] . \end{aligned}$$

The upper bound requires slightly more attention. Note that, by the choice of *m*, $\log m\ll x_1\vartheta$. Therefore $\log m\le x_i\vartheta$ for all large *n*. Hence, for all large *n*,

$$\begin{aligned} \Delta \pi (x_i\vartheta ) <\mathbf{P}(\mathrm{Bin}(x_{i+1}\vartheta ,p)>\log m) +\sum _{\ell =r}^{\log m}\Delta \mathbf{P}(\mathrm{Bin}(x_{i}\vartheta ,p)=\ell ). \end{aligned}$$

Since $p\vartheta \ll 1\ll m$, for all large *n*,

$$\begin{aligned} \mathbf{P}(\mathrm{Bin}(x_{i+1}\vartheta ,p)> \log m) \le \vartheta (x_{i+1}p\vartheta )^{1+\log m}. \end{aligned}$$

Therefore, by (1), (19) and the choice of *m*,

$$\begin{aligned} \frac{rn}{\vartheta \Delta (x_{i}^r)} \mathbf{P}\left( \mathrm{Bin}(x_{i+1}\vartheta ,p)> \log m\right) \le O\left( \frac{n}{\Delta x_i}(p\vartheta )^{1+\log m}\right) \ll p\vartheta . \end{aligned}$$

Next, by (19), it follows that, for all $\ell \le \log m$ and large *n*,

$$\begin{aligned} \Delta \mathbf{P}(\mathrm{Bin}(x_{i}\vartheta ,p)=\ell )&\le \frac{(p\vartheta )^\ell }{\ell !}\left[ x_{i+1}^\ell -x_i^\ell \left( 1-\frac{\ell }{x_i\vartheta }\right) ^\ell \right] \\&\le \frac{(p\vartheta )^\ell }{\ell !}\Delta (x_i^\ell )\left( 1 +\frac{\ell }{\vartheta \Delta x_i}\right) . \end{aligned}$$

Therefore, by (1) and (19), for all large *n*,

$$\begin{aligned} \frac{rn}{\vartheta }\sum _{\ell =r}^{\log m}\Delta \mathbf{P}(\mathrm{Bin}(x_{i}\vartheta ,p)=\ell )&\le \sum _{\ell =r}^{\log m}\frac{r!(p\vartheta )^{\ell -r}}{\ell !}\Delta (x_i^\ell ) \left( 1+\frac{\ell }{\vartheta \Delta x_i}\right) \\&\le \Delta (x_i^r) \left( 1+\frac{\log m}{\vartheta \Delta x_i}\right) \left[ 1 +\sum _{\ell >0}O(p\vartheta )^\ell \right] \\&\le \Delta (x_i^r) \left[ 1+O\left( p\vartheta +\frac{1}{\log \vartheta }\right) \right] . \end{aligned}$$

Altogether, we find that

$$\begin{aligned} \frac{rn}{\vartheta }\Delta \pi (x_i\vartheta ) = \Delta (x_i^r)\left[ 1+O\left( p\vartheta +\frac{1}{\log \vartheta }\right) \right] \end{aligned}$$

as claimed. $$\square $$
